# Clinico-Genomic Analysis Reiterates Mild Symptoms Post-vaccination Breakthrough: Should We Focus on Low-Frequency Mutations?

**DOI:** 10.3389/fmicb.2022.763169

**Published:** 2022-03-03

**Authors:** Akshay Kanakan, Priyanka Mehta, Priti Devi, Sheeba Saifi, Aparna Swaminathan, Ranjeet Maurya, Partha Chattopadhyay, Bansidhar Tarai, Poonam Das, Vinita Jha, Sandeep Budhiraja, Rajesh Pandey

**Affiliations:** ^1^INtegrative GENomics of HOst-PathogEn (INGEN-HOPE) Laboratory, CSIR-Institute of Genomics and Integrative Biology (CSIR-IGIB), Delhi, India; ^2^Academy of Scientific and Innovative Research (AcSIR), Ghaziabad, India; ^3^Max Super Speciality Hospital (A Unit of Devki Devi Foundation), Max Healthcare, Delhi, India

**Keywords:** COVID-19, vaccination breakthrough, clinico-genomic, low-frequency mutations, disease severity, integrative analysis

## Abstract

Vaccine development against severe acute respiratory syndrome coronavirus 2 (SARS-CoV-2) has been of primary importance to contain the ongoing global pandemic. However, studies have demonstrated that vaccine effectiveness is reduced and the immune response is evaded by variants of concern (VOCs), which include Alpha, Beta, Delta, and, the most recent, Omicron. Subsequently, several vaccine breakthrough (VBT) infections have been reported among healthcare workers (HCWs) due to their prolonged exposure to viruses at healthcare facilities. We conducted a clinico-genomic study of ChAdOx1 (Covishield) VBT cases in HCWs after complete vaccination. Based on the clinical data analysis, most of the cases were categorized as mild, with minimal healthcare support requirements. These patients were divided into two sub-phenotypes based on symptoms: mild and mild plus. Statistical analysis showed a significant correlation of specific clinical parameters with VBT sub-phenotypes. Viral genomic sequence analysis of VBT cases revealed a spectrum of high- and low-frequency mutations. More in-depth analysis revealed the presence of low-frequency mutations within the functionally important regions of SARS-CoV-2 genomes. Emphasizing the potential benefits of surveillance, low-frequency mutations, D144H in the *N* gene and D138Y in the *S* gene, were observed to potentially alter the protein secondary structure with possible influence on viral characteristics. Substantiated by the literature, our study highlights the importance of integrative analysis of pathogen genomic and clinical data to offer insights into low-frequency mutations that could be a modulator of VBT infections.

## Introduction

The coronavirus disease 2019 (COVID-19) pandemic, now leading to more than 272 million confirmed infections worldwide and with over a billion estimated infections, has made the acquisition of herd immunity a vital measure to halt viral transmission (Shastri et al., 2021b). Accelerating this endeavor by the rapid development of vaccines has thereby been a priority that has been progressing at a remarkable pace around the globe. By early December 2020, the first approved vaccines were available for public use (Kanakan et al., 2020; Andreano and Rappuoli, 2021). Although the vaccines showed good efficacy in clinical trials (Kyriakidis et al., 2021), their safety and effectiveness in a real community setting are equally important aspects. Several studies highlighting these aspects have been published (Butt et al., 2021; Daniel et al., 2021; Haas et al., 2021). Among individuals vaccinated between 8 December 2020 and 10 March 2021 in the United Kingdom, a significant reduction in infection risk was seen after 12 days of vaccination (BNT162b2/Pfizer and ChAdOx1/Covishield), with less frequent side effects as compared to phase three clinical trials (Menni et al., 2021).

Viral genome sequencing of the breakthrough cases has enabled us to understand the association of severe acute respiratory syndrome coronavirus 2 (SARS-CoV-2) variants with reduced vaccine efficacy. Subsequently, effectiveness was found to be relatively decreased against new variants of concern (VOCs) (Kustin et al., 2021). One such VOC, B.1.617.2, also known as the Delta variant and first identified in India in October 2020 (WHO, 2021), is characterized by mutations T19R, G142D, Δ157–158, R158G, L452, T478K, D614G, P681R, and D950N in the spike protein (Planas et al., 2021). The key mutations, L452R and T478K, in the receptor-binding domain (RBD) of the spike protein have been associated with higher transmission rates as they enhance the binding to the ACE2 receptor (Lazarevic et al., 2021). The Delta variant has been reported to be 60% more transmissible than the Alpha (B.1.1.7) variant (Callaway, 2021) and was responsible for the massive increase in COVID-19 cases in India during the second surge of infections between April and May 2021.

Mutations that alter the characteristics of the RBD in the spike protein, such as L452R and T478K in the Delta variant, can affect antibody neutralization (Planas et al., 2021). Hence, it is crucial to evaluate the efficiency of the neutralizing antibody against the Delta variant in vaccinated individuals following administration of the first and second doses. Gajanan et al. demonstrated 3.2- to 4.5-fold lower neutralizing antibody titer values in the sera of Covishield-vaccinated individuals infected with Delta than with B.1 variant-infected individuals (Sapkal et al., 2021; Wall et al., 2021). Also, Covishield was reported to elicit 2.5-fold lower neutralizing antibody (NAb) titers than Pfizer against Delta after two doses (Wall et al., 2021). Consequently, the reduction in NAb titer values contributes to reduced vaccine effectiveness among vaccinated individuals. Various studies on vaccine breakthrough (VBT) infections in healthcare workers (HCWs) have been reported so far (Bergwerk et al., 2021; Geysels et al., 2021; Ma et al., 2021; Philomina et al., 2021; Tyagi et al., 2021), unsurprisingly due to their prolonged exposure to the virus compared to the general public (Rudberg et al., 2020). Although robust efforts to limit hospital-acquired infections have been undertaken, high rates of nosocomial infections (Lumley et al., 2021) and seroconversions were seen among HCWs (Shah et al., 2020). The clinical severity of these breakthrough infection cases is an aspect that merits detailed investigation. Current studies have associated VBT infections with majority mild and a subset with moderate clinical presentations (Estofolete et al., 2021; Rovida et al., 2021; Teran et al., 2021), as well as limited case reports of severe infection (Ioannou et al., 2020; Shastri et al., 2021a; Brosh-Nissimov et al., 2021). A study by Fransesca et al. on HCWs has reported VBT infections as being mostly mildly symptomatic or asymptomatic with a lower transmission rate (Rovida et al., 2021). However, Tal Brosh et al. reported that individuals with comorbidities presented severe symptoms upon contracting VBT (BNT162b2) infections. However, the clinical profiles of other VBT-infected patients almost resembled those of unvaccinated individuals (Brosh-Nissimov et al., 2021), thereby demonstrating the need to explore the nature of VBT infections and their association with SARS-CoV-2 variants, which could reflect on the effectiveness of a vaccine.

Our study findings reiterate: (i) the strength of clinico-genomics co-analysis, which captures the clinical data and SARS-CoV-2 genome sequencing; (ii) the spectrum of mutations in the disease sub-phenotypes; (iii) functional role of mutations; (iv) the role of mutations in protein structure modulation; and (v) highlighting the importance of genomic surveillance of low-frequency mutations for their future potential role in immune escape. Given that VBTs across different vaccines are a reality, albeit with milder symptoms, it would be important to closely monitor the low-frequency mutations vis-a-vis their possible role in immune escape and new evolving SARS-CoV-2 VOCs and variants of interest (VOIs).

## Materials and Methods

### Clinical Data

The patient clinical data of 74 COVID-19-positive, fully vaccinated (Covishield) HCWs were retrospectively collected from Max Super Speciality Hospital, Saket, New Delhi, India, and anonymized at the data warehouse of CSIR-IGIB. The patients selected for the study were confirmed COVID-19 positive by SARS-CoV-2-specific RT-PCR. The electronic patient records inclusive of patient demography, clinical symptoms at the time of admission, comorbidities, high-resolution computed tomography (HRCT) scores, and the medications administered to the patients were analyzed.

### Patient Severity Classification

To closely observe the differences in VBT infections at the subclinical level, the patients were segregated into two groups with relative differences in disease severity. Data pertaining to patient symptoms, HRCT scores, and antiviral administration were considered for this classification using the hereby described scoring system. Any symptoms reported by the patients were scored as 1 and no symptoms scored as 0. A comparatively high HRCT score (>3) and antiviral administration were scored as 2. Patients with a total score ≤ 2 were grouped as “mild,” thereby exhibiting fewer clinical presentations in the disease period. The rest of the patients with a total score ≥ 3 were classified as “mild plus,” exhibiting comparatively more clinical presentations and thereby requiring targeted medication in the disease period.

### qRT-PCR

Viral RNA was extracted from viral transport medium (VTM) solution or liquefied sputum samples using a commercially available RNA extraction kit (QIAmp viral mini kit, cat. no 52906; Qiagen, Hilden, Germany). Of the VTM solution or liquefied sputum, 200 μl was processed for lysing and viral enrichment, in accordance with the protocol in the kit (QIAamp Viral RNA Mini Handbook). After washing with the wash buffers, viral RNA was eluted in RNase-free water. qRT-PCR for SARS-CoV-2 detection was performed using the TRUPCR SARS-CoV-2 kit (cat. no 3B304; 3B BlackBio Biotech India Ltd., Bhopal, India). Briefly, 10 μl RNA was added to 15 μl of the reaction mixture in accordance with the protocol in the kit. The RT-PCR assay was run on Rotor-Gene Q (Qiagen) for the SARS-CoV-2 genes envelope (*E*), nucleocapsid (*N*), and RdRp, with the RNaseP gene (human) as the internal positive control. Initially, viral RNA was converted to complementary DNA (cDNA) by reverse transcription at 50°C for 5 min with the TRUPCR enzyme mix. The resultant cDNA copies were further amplified using the following conditions: initial denaturation at 95°C for 5 min, denaturation at 95°C for 5 s, annealing at 60°C for 45 s, and extension at 72°C for 15 s defined for a total of 40 cycles. However, for the interpretation of results, a cycle threshold cutoff of 35 was considered.

### Statistical Analysis

The clinical data were summarized using descriptive statistics, wherein continuous variables were represented as the median and interquartile range (IQR) and the categorical variables represented as percentages (*n*, %). We used the chi-square test to compare categorical variables and the Mann–Whitney *U* test to compare continuous variables. A *p*-value of < 0.05 was considered significant.

### SARS-CoV-2 Whole-Genome Sequencing

In brief, 100 ng of RNA was used for double-stranded cDNA synthesis. This involves first-strand cDNA synthesis using Superscript IV (cat. no. 18091050; Thermo Fisher Scientific, Waltham, MA, United States), followed by RNase H digestion of single-stranded RNA and second-strand synthesis by DNA polymerase I, large (Klenow) fragment (cat. no. M0210S; New England Biolabs, Ipswich, MA, United States). The double-stranded cDNA thus obtained was purified using AMPure XP beads (cat. no. A63881; Beckman Coulter, Brea, CA, United States). The SARS-CoV-2 genome was then amplified from 100 ng of the purified cDNA with the ARTIC V3 primer protocol. For sequencing library preparation using Oxford Nanopore Technology (ONT), preparation consisted of end repair/dA tailing, native barcode ligation, and adapter ligation. This was performed with 200 ng of the multiplexed PCR amplicons according to the ONT library preparation protocol—PCR Tiling of COVID-19 Virus (version: PTC_9096_v109revE_06Feb2020). Sequencing in sets of 24 barcoded samples was performed on the MinION Mk1C platform.

Sequencing library preparation for Illumina was performed using 100 ng of purified ARTIC PCR product using the Illumina DNA Prep kit (cat. no. 20018705; Illumina, San Diego, CA, United States). The process involved tagmentation, followed by post-tagmentation cleanup and amplification by PCR leading to indexed DNA fragments, which was purified prior to sequencing. The quality and quantity of the sequencing library were checked using an Agilent 2100 Bioanalyzer with high-sensitivity DNA chip and the Qubit dsDNA HS Assay Kit, respectively. A loading concentration of 11 pM was prepared by denaturing and diluting the libraries in accordance with the MiSeq System Denature and Dilute Libraries Guide (document no. 15039740 v10; Illumina). Sequencing was performed on MiSeq using the MiSeq Reagent Kit v3 (150 cycles) at a read length of 2 × 75 bp.

### Sequencing Data Analysis

The ARTIC end-to-end pipeline ARTIC (2021) was used for the analysis of MinION raw fast5 files up to the variant calling. Raw fast5 files of the samples were base called and demultiplexed using Guppy basecaller, which uses the base calling algorithms of Oxford Nanopore Technologies (Nanopore Community, 2021) with a Phred quality cutoff score > 7 on a GPU-Linux accelerated computing machine. Reads with a Phred quality score of less than 7 were discarded to filter the low-quality reads. The resultant demultiplexed fastq were normalized by a read length of 300–500 (approximate size of amplicons) for further downstream analysis and aligned to the SARS-CoV-2 reference (MN908947.3) using the aligner Minimap2 v2.17 (Li, 2018). Nanopolish (Loman et al., 2015) was used to index raw fast5 files for variant calling from the minimap output files. To create consensus fasta, bcftools v1.8 was used over the normalized minimap2 output.

Fastqc was performed for all the raw fastq files generated from Illumina sequencing in order to check the Phred quality scores of all the sequences (FastQC: A Quality Control Tool for High Throughput Sequence Data, Babraham Bioinformatics, 2021a). A Phred quality score threshold of > 20 was used for filtering reads from all the samples. Subsequently, adapter trimming was performed using the Trim Galore tool (Trim Galore!, Babraham Bioinformatics, 2021b), and alignment of the sequences was performed using the HISAT2 algorithm (Kim et al., 2019) on human data build hg38 to remove any human read contamination. BEDTools was used to generate the consensus fasta using the unaligned/filtered reads, and variant calling was performed using the high-quality reads (Quinlan and Hall, 2010). The sequencing depth and genome coverage for all the samples are available in Supplementary Table 1.

### Phylogenetic Analysis

The Wuhan reference genome for SARS-CoV-2 (NC_045512.2) was used to perform multiple sequence alignment of the 56 genome sequences of SARS-CoV-2 with minimum coverage of more than 50% using MAFFT (v7.475) (Katoh et al., 2002). Alignment was manually trimmed and a phylogenetic tree was constructed using the IQ-tree (Nguyen et al., 2015). Lineage classification was performed using PANGOLIN (Rambaut et al., 2020). The phylogenetic analysis was visualized using FIGTREE software (FigTree, 2021).

### Mutation Analysis

From the variants called with high-quality score in the vcf file, the high- and low-frequency synonymous and non-synonymous mutations in the samples were extracted and a heatmap was generated using the Heatmapper tool (http://www.heatmapper.ca/). A lollipop plot representing low-frequency (<10% of patients) and high-frequency (>45% of patients) mutations was generated in R using the packages g3, viz., (Guo, 2021), rtracklayer (Michael Lawrence, 2017), and trackViewer (Ou and Zhu, 2019), followed by data visualization using the ggplot2 package (ggplot2, 2021). Furthermore, global frequency of the mutations was checked against a global dataset available at 2019 Novel Coronavirus Resource (2019nCoVR), CNCB (Song et al., 2020), with 21,51,254 sequences as of October 2021.

### Structure Analysis

To understand the effect of mutations on the protein, we performed primary and secondary structural analysis of the SARS-CoV-2 proteins. SARS-CoV-2 reference sequences (NC_045512) for the spike and nucleocapsid proteins were downloaded from NCBI. To study the effect of mutation on the physiochemical properties of the peptides, such as net charge at neutral pH, extinction coefficient, and isoelectric point, we used Innovagen’s peptide calculator (INNOVAGEN, 2021). To further observe the effect of mutations on the folding and secondary structure of the peptides, we used the CFSSP tool (Ashok Kumar, 2013).

## Results

### Clinical Characteristics of COVID-19 Patients

In our patient cohort, it was distinctly observed that none of the patients succumbed to the infection, nor did any of them require intensive medical care or respiratory support. Considering the diversity of the patient demographics in our study, this observation can be a direct result of vaccine-acquired immunity against SARS-CoV-2 in our patients. After sub-classification, 31 patients were grouped into the mild category and 42 patients into the mild plus category. The statistical correlation analysis of the parameters not considered for patient sub-grouping showed a few clinical parameters correlating with our classification, these being duration of symptom presentation (*p* = 0.047), anticoagulant administration (*p* = 0.029), and ivermectin administration (*p* = 0.04) (Table 1).

**TABLE 1:** Clinical summary of the severe acute respiratory syndrome coronavirus 2 (SARS-CoV-2)-positive vaccinated patients.

Parameter	Total (*N* = 73)	Mild (*n* = 31)	Mild plus (*n* = 42)	*p*-Value
Age (years)	42 (34–51)	40 (36–53)	42.5 (33.25–51)	0.984*^a^*
Gender (F/M)	29/44	12/19	17/25	0.87*^b^*
**Signs and symptoms**				
Fever	49 (67.12%)	13 (41.93%)	36 (85.71%)	** <0.001*^b^***
Cough	33 (45.20%)	10 (32.25%)	23 (54.76%)	0.056*^b^*
Sore throat	24 (32.87%)	7 (22.58%)	17 (40.47%)	0.107*^b^*
Headache	17 (23.28%)	4 (12.90%)	13 (30.95%)	0.07*^b^*
Bodyache	28 (38.35%)	3 (9.67%)	25 (59.52%)	** <0.001*^b^***
Breathing difficulty	6 (8.12%)	1 (3.22%)	5 (11.90%)	0.182*^b^*
Loss of taste or smell	10 (13.69%)	1 (3.22%)	9 (21.42%)	**0.025*^b^***
Others (weakness or diarrhea)	33 (45.20%)	11 (35.48%)	22 (52.38)	0.151*^b^*
Hospitalized	28 (38.35%)	9 (29.03%)	19 (45.23%)	0.159*^b^*
Length of stay (days)	7 (6–9)	8 (7–9)	6.5 (5.25–9)	0.423*^a^*
Home quarantine	45 (61.64%)	22 (70.97%)	23 (54.77%)	0.159*^b^*
Duration of symptom presentation	4 (3–6)	3 (2–5)	5 (3–6)	**0.047*^a^***
Comorbidities	14 (19.17%)	5 (16.12%)	9 (21.42%)	0.569*^b^*
No comorbidities	59 (80.82%)	26 (83.87%)	33 (78.57%)	0.569*^b^*
**Treatment/antivirals**				
Favipiravir	35 (47.94%)	12 (41.93%)	23 (54.76%)	0.174*^b^*
Remdesivir	7 (9.58%)	1 (3.22%)	6 (14.28%)	0.112*^b^*
Ivermectin	28 (38.35%)	16 (51.64%)	12 (28.57%)	**0.04*^b^***
Antibiotics	37 (50.68%)	16 (51.64%)	21 (50%)	0.891*^b^*
Steroids	20 (27.39%)	5 (16.12%)	15 (35.71%)	0.063*^b^*
Anticoagulants	13 (17.80%)	2 (6.45%)	11 (26.19%)	**0.029*^b^***
*C*_*t*_ value (RdRp)	18.03 (16.38–20.79)	18.03 (15.93–18.47)	18.03 (16.95–21.03)	0.207*^a^*

*Data shown are the median (IQR) or n (%).*

*^a^Mann–Whitney U test.*

*^b^Chi-square test. Significant p values are highlighted in bold.*

### Phylogenetic Analysis of SARS-CoV-2

**FIGURE 1:**
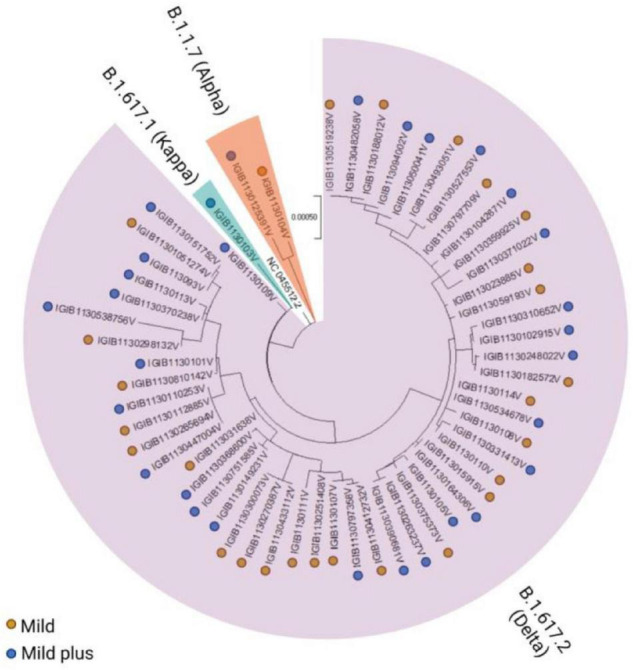
Phylogenetic distribution of severe acute respiratory syndrome coronavirus 2 (SARS-CoV-2) variants across the mild and mild plus categories of coronavirus disease 2019 (COVID-19) patients. The vaccination breakthroughs primarily by the Delta variant, which led to the second surge in India from April to May 2021, are highlighted.

Viral whole-genome sequencing data were used to observe the phylogenetic differences in the VBT viral sequences. Figure 1 shows the phylogenetic and severity distributions across patient samples with high genome coverage (*n* = 56). We observed that more than 90% of the samples (*n* = 51) belonged to the B.1.617.2 (Delta) lineage, less than 5% of the samples belonged to the B.1.1.7 (Alpha) lineage, and only one sample belonged to the B.1.617.1 (Kappa) lineage. The abundance of patients in the mild and mild plus categories was observed to be consistent across all lineages.

### Mutation Analysis of the Vaccine Breakthrough Infections

**FIGURE 2:**
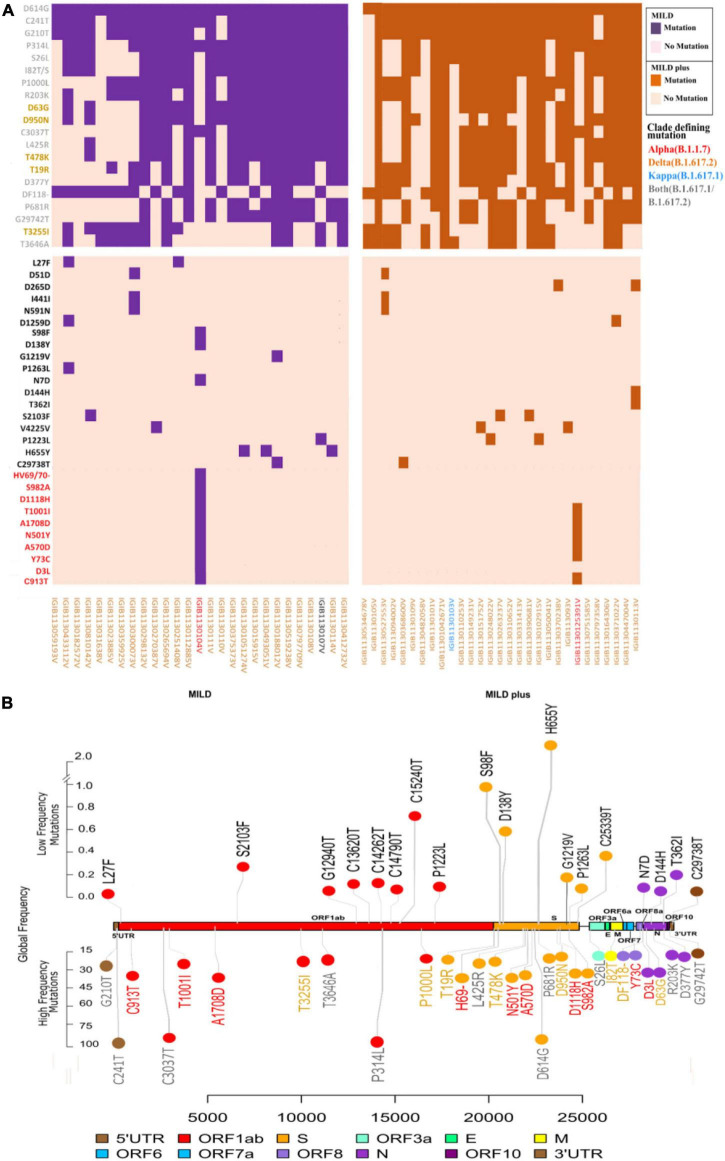
Spectrum of high- and low-frequency mutations observed across the severe acute respiratory syndrome coronavirus 2 (SARS-CoV-2) genomes. (**A**) Integrative heatmap representing the top high-frequency and low-frequency mutations in clinical sub-phenotypes: mild and mild plus. Sample IDs are colored to represent the lineage of each sample. (**B**) The *upper panel* shows the location of the top 18 mutations with global low frequency, while the *lower panel* shows the global high frequency and the clade-defining mutations of variants of concern (VOCs)/variants of interest (VOIs) in the SARS-CoV-2 genomes. Lineage-defining mutations for the different variants detected are marked in different colors: *red* for Alpha (B.1.1.7), *orange* for Delta (B.1.617.2), and *blue* for Kappa (B.1.617.1).

A comprehensive analysis of all mutations present in the dataset showed the presence of 119 unique mutations. Further segregation of the mutations as high frequency (present in > 45% of patients) gave a set of 20 mutations. Segregation as low frequency, observed in less than 10% of the samples, showed the presence of 78 mutations, with the majority of 61 mutations only observed in any one sample. However, we ensured that there was high sequencing depth for that mutation to have high confidence on the mutation(s). Subsequently, we looked into the global presence of the identified mutations. We investigated the frequency of these mutations from a database of the concurrent time with 21,51,254 samples from different parts of the world. Upon performing global frequency analysis, it was observed that all the high-frequency mutations in our cohort were highly frequent globally (>20% of sequences) and belonged to the set of clade-defining mutations of the SARS-CoV-2 variant B.1.617.2 (Delta) (Table 2). This is unsurprising as almost all of our patients were found to be infected with this variant (Delta), and these mutations, being highly frequent in the global population, have been reported and studied by various groups throughout the world (Supplementary Table 2). To understand, explore, and elucidate the mutations with potential significance toward functional modulation of SARS-CoV-2 and thereby warrant continued surveillance, we looked at the set of low-frequency mutations in our cohort. Ensuring the high quality (depth > 100) of these mutations and detection in at least two patient samples, we noted 17 unique mutations. To increase this pool of potentially functional background mutations in SARS-CoV-2, we added all 11 high-quality structural mutations from the large pool of 61 mutations present in any one patient as well. Global frequency analysis of the 28 low-frequency mutation set from our cohort showed that all the mutations, except for the 10 clade-defining mutations of the Alpha (B.1.1.7) variant from two patients, were present in less than 1.5% global sequences. To obtain a final set of background mutations, we removed these low-frequency mutations with high global frequency from the list of low-frequency mutations, thus generating a list of 18 unique mutations present in low frequencies globally and in our cohort. The cohort frequency, global frequency, genomic coordinates, and the lineage association of these high- and low-frequency mutations are presented graphically in Figure 2.

**TABLE 2:** High- and low-frequency mutations of SARS-CoV-2 with vaccine breakthrough (VBT) cohort frequency, global frequency, and mutations belonging to different lineages.

Nucleotide change	Amino acid change	Cohort frequency (%) (*n* = 56 patients)	Global frequency (%) (*n* = 21,51,254 samples)	Lineage to which mutations belong
**A23403G**	S:D614G	100	99.02912	B.1.617.1, B.1.617.2, B.1.1.7
**C241T**	5′UTR	92.85714	98.86285	B.1.617.1, B.1.617.2, B.1.1.7
**G210T**	5′UTR	91.07143	27.02238	B.1.617.1, B.1.617.2
**C14408T**	ORF1b:P314L	83.92857	98.90283	B.1.617.1, B.1.617.2, B.1.1.7
**C25469T**	ORF3a:S26L	78.57143	26.76616	B.1.617.1, B.1.617.2
**T26767C**	M:I82T/S	76.78571	27.15128	B.1.617.1, B.1.617.2
**C16466T**	ORF1b:P1000L	75	26.31028	B.1.617.2
**G28881T**	N:R203K	73.21429	26.82487	B.1.617.2, B.1.1.7
**A28461G**	N:D63G	69.64286	25.79389	B.1.617.2
**G24410A**	S:D950N	67.85714	25.70185	B.1.617.2
**C3037T**	ORF1a:924	66.07143	98.92783	B.1.617.2
**T22917G**	S:L425R	66.07143	28.66272	B.1.617.2
**C22995A**	S:T478K	66.07143	27.268	B.1.617.2
**C21618G**	S:T19R	64.28571	26.55707	B.1.617.2
**G29402T**	N:D377Y	60.71429	27.69152	B.1.617.2
**AGATTTC28247A**	ORF8:DF118-	58.92857	25.61143	B.1.617.2
**C23604G**	S:P681R	53.57143	26.97538	B.1.617.2
**G29742T**	3′UTR	51.78571	25.91149	B.1.617.2
**C10029T**	ORF1a:T3255I	48.21429	22.69211	B.1.617.2
**A11201G**	ORF1a:T3646A	46.42857	21.78706	B.1.617.1, B.1.617.2
C17135T	ORF1b:P1223L	5.357143	0.143451	B.1.1.7
C23525T	S:H655Y	5.357143	2.258915	B.1.1.7
C6573T	ORF1a:S2103F	5.357143	0.363974	B.1.1.7
G12940T	ORF1a:V4225V	5.357143	0.008786	B.1.1.7
C344T	ORF1a:L27F	3.571429	0.049413	B.1.1.7
**C913T**	ORF1a:C913T	3.571429	38.06138	B.1.1.7
C13620T	ORF1b:D51D	3.571429	0.141452	B.1.1.7
C14262T	ORF1b:D265D	3.571429	0.140709	B.1.1.7
C14790T	ORF1b:I441I	3.571429	0.09018	B.1.1.7
C15240T	ORF1b:N591N	3.571429	0.706193	B.1.1.7
C25339T	S:D1259D	3.571429	0.490644	B.1.617.2
C29738T	3′UTR	3.571429	0.17441	B.1.617.2
**A28111G**	ORF8:Y73C	3.571429	38.20432	B.1.617.2
**A23063T**	S:N501Y	3.571429	41.18254	B.1.617.2
**C3267T**	ORF1a:T1001I	3.571429	38.29134	B.1.617.2
**C5388A**	ORF1a:A1708D	3.571429	38.2132	B.1.617.2
**C23271A**	S:A570D	3.571429	38.19219	B.1.1.7
C21855T	S:S98F	1.785714	1.349585	B.1.1.7
G21974C	S:D138Y	1.785714	0.60151	B.1.617.2
G25218T	S:G1219V	1.785714	0.206763	B.1.617.2
A28295G	N:N7D	1.785714	0.149029	B.1.1.7
G28703T	N:D144H	1.785714	0.080883	B.1.617.2
C25350T	S:P1263L	1.785714	0.100546	B.1.617.2
C29358T	N:T362I	1.785714	0.223637	B.1.617.2
**G28280C**	N:D3L	1.785714	38.01202	B.1.617.2
**ATACATG21764A**	S:HV69/70-	1.785714	38.65676	B.1.617.2, B.1.1
**T24506G**	S:S982A	1.785714	38.18898	B.1.617.2
**G24914C**	S:D1118H	1.785714	38.17587	B.1.617.2

*Mutations in bold are clade-defining mutations.*

### Low-Frequency Mutations in Vaccine Breakthrough and Their Potential Global Significance

As almost all patients showed mild symptoms, we hypothesized whether the low-frequency mutations hold any clue toward the present breakthrough infections and potential future importance. Thus, we further studied the low-frequency mutational spectra among all patients and the global database to understand whether breakthrough infections can be associated with immune escape mutations. Our set of 18 background mutations hereby took into account global and cohort low frequencies and structural mutants of the very low-frequency mutation cohort (only present in one patient). Varied distributions of these mutations throughout the SARS-CoV-2 genome were observed, with an overrepresentation of six mutations in the spike protein, five in ORF1b, three mutations in ORF1a and the *N* gene, and one in 3′-UTR.

A comprehensive literature review of all the detected mutations showed that mutations with high global frequency have been studied more frequently and that the globally reported low-frequency mutations have been sparsely reported and less studied (Supplementary Table 2). This selection of mutations is understandable from a viral transmission standpoint, wherein a mutation conferring improved transmission characteristics would be highly frequent in a global cohort. But at the same time, it may fall short in understanding the comprehensive group of mutations leading to vaccination breakthroughs as, at this point, only a small fraction of the global population has been vaccinated with regional variability. Therefore, viral genomic mutations, when observed as a collective set of mutations irrespective of their origin from a breakthrough or non-breakthrough infection, can hinder the identification of mutations that may be leading to the vaccination breakthrough.

We observed only a few globally low-frequency mutations that were previously reported and fewer being analyzed for their structural and functional effects. Notably, the low-frequency mutation H655Y was reported to naturally evolve in SARS-CoV-2 in positive selection and has been reported in multiple cohorts (Dieterle et al., 2020; Braun et al., 2021; Szemiel et al., 2021). Mutation D138Y has been associated with a decrease in neutralizing monoclonal antibodies (Wang et al., 2021). One study reported that mutation P1263L was associated with decreased transmission of the virus (Li et al., 2020; Qi et al., 2021) A study also reported a stabilizing effect of the mutation T362I using *in silico* studies (Rahman et al., 2021).

### Peptide Sequence and Structure Analysis of Low-Frequency Mutations

**FIGURE 3:**
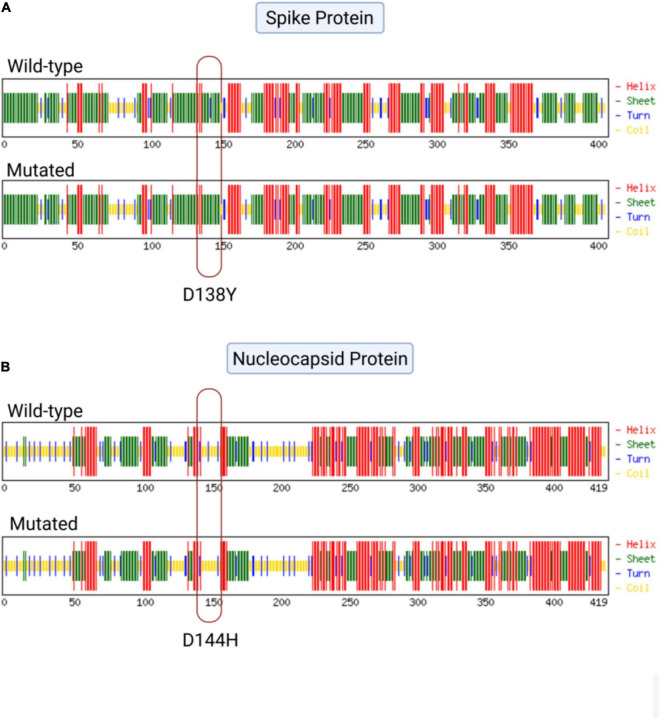
Effect of mutations on the secondary structure of the spike and nucleocapsid proteins. (**A**) Mutation D138Y in the spike protein of severe acute respiratory syndrome coronavirus 2 (SARS-CoV-2) showing the conversion of turn (*blue*) into sheet (*green*). (**B**) Mutation D144H in the nucleocapsid protein of SARS-CoV-2 showing the conversion of turn (*blue*) into coil (*yellow*).

To observe the structural changes in SARS-CoV-2 due to low-frequency mutations, we first observed the changes in the physiochemical properties of the structural proteins due to the mutations. We observed that the D144H and N7D mutations in the nucleocapsid region and the D138Y and H655Y mutations in the spike protein showed changes in extinction coefficient and net charge at pH 7 (*Z*_*ce*_) (Supplementary Table 3). To further evaluate the effect of these mutations, we performed a secondary structure analysis. We observed that two low-frequency mutations, D138Y in the spike protein and D144H in the nucleocapsid protein, showed secondary structural changes. The conversion of aspartic acid into tyrosine at position 138 in the spike protein showed the conversion of turn in the wild type to sheet in the mutant protein (Figure 3A). Similarly, mutation of aspartic acid into histidine at position 144 in the nucleocapsid protein resulted in the formation of coil at the mutant location instead of the turn observed in the wild type (Figure 3B).

## Discussion

Global cooperation is toward developing a COVID-19 vaccine that is both safe and effective. However, the recently emerged SARS-CoV-2 VOCs have resulted in breakthrough infections after the vaccine regimen has been completed (Sapkal et al., 2021). Numerous studies have recently reported on VBT infections by VOCs and the reduced effectiveness of vaccines in antibody neutralization studies (CDC COVID-19 Vaccine Breakthrough Case Investigations Team, 2021; Farinholt et al., 2021; Ioannou et al., 2021; Philomina et al., 2021). At the same time, it also highlighted the mild symptoms in the majority of VBT studies. HCWs, being frontline workers, were more vulnerable to contract the virus due to their elongated exposure and interactions with hospitalized infected patients. Although consensus guidelines are followed in healthcare facilities, nosocomial infections have been highly reported among HCWs as compared to community-acquired infections (Rudberg et al., 2020). Observational studies on VBT infections in HCWs can reflect the need for improved strategies and facilities required in hospitals to restrain nosocomial infections.

Our study reemphasized the mild clinical severity of VBT infections in HCWs, but at the same time highlighted the potential importance of low-frequency mutations, as evidenced by the structural outcomes of selective mutations observed among VBT-infected individuals (Figure 3). The cohort consisted of 74 HCWs of variable ages, fully vaccinated with Covishield, who tested positive for SARS-CoV-2 by RT-PCR. The disease severity in VBT infections did not show an association with SARS-CoV-2 mutations, with the possible role of host genetic and immunological parameters involved in COVID-19 response (Fung and Liu, 2019; Maurya et al., 2021; Pairo-Castineira et al., 2021). The involvement of host parameters in modulating vaccine-driven immunological memory response toward SARS-CoV-2 is a subject for future studies.

Our analysis toward observing specific mutations in our samples revealed the presence of potentially functional mutations. Such mutations can be classified as high- and low-frequency mutations based on their occurrence in the sample compared with the global datasets (Table 2). All the mutations observed in higher frequencies were well studied due to their stability and global distribution (Cele et al., 2021; Collier et al., 2021; Yi et al., 2021), with some of them being used as lineage-defining mutations (Dhar et al., 2021; Lauring and Hodcroft, 2021; Tegally et al., 2021; Figure 2). Understanding and reporting low-frequency mutations with potential implications in functional modulation is a valuable resource for rapid strain containment response after performing validation studies (Pandey and Agrawal, 2020). We identified a few such low-frequency mutations previously reported with VBT infections (D138Y and H655Y) (Estofolete et al., 2021). The mutation D138Y and four other mutations in the S protein have been reported to change the surface potential of the N-terminal domain (NTD) in protein modeling studies (Fantini et al., 2021). Wang et al. also observed the reduced efficacy of the NTD anti-SARS-CoV-2 antibody against the D138Y mutant spike protein. Moreover, the mutation R203K/G204R was found to display a different protein structure morphology from the control and an enhanced intraviral interaction of R203K/G204R in the N and E proteins in molecular docking and protein structure prediction analysis (Wu S. et al., 2021). In a preprint manuscript, this mutation in the N protein was reported to increase the infectivity, fitness, and virulence of SARS-CoV-2 (Wu H. et al., 2021). Based on the observed potential of the low-frequency mutations determined in our study, further investigations of low-frequency mutations and their plausible role in modulating the protein structure are expected to enhance our understanding.

**FIGURE 4:**
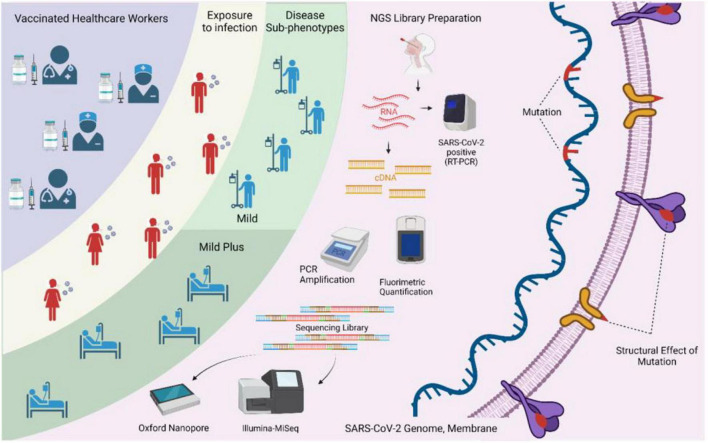
Study design and methodology used in the study. The integrative aspects of surveillance of healthcare workers, vaccination, breakthrough infections, sequencing-based identification of severe acute respiratory syndrome coronavirus 2 (SARS-CoV-2) genome information, and elucidation of the possible functional role of the low-frequency mutations are highlighted.

We also performed primary and secondary *in silico* structural analyses of the low-frequency mutations in the SARS-CoV-2 structural genes observed in our study to elucidate the functional significance of the variants, if any. Herein, the mutations D138Y in the spike protein and D144H in the nucleocapsid protein were observed to significantly change the protein folding dynamics, thereby influencing secondary structure formation (Figure 3). As a result, these mutations have potential in function modulation, which requires further investigation using *in vitro* techniques to understand their role in immune evasion and increased transmission in order to classify these variants based on the risk they pose to public health. The highlights of our study design and analysis have been captured in a graphical format in Figure 4.

As globally, inclusive of India, we are moving with a larger fraction of the population receiving vaccines. It has been satisfying to observe mild symptoms in breakthrough infections. At the same time, genomic surveillance is required and is important to discover and detect the mutations, especially the low-frequency ones during breakthrough infections, at a given time. These low-frequency mutations may be important, especially by virtue of their presence in the important functional domains of SARS-CoV-2. Whether or not they are causal or modulators of breakthrough infections is a matter of future investigation. This study is timely as we may be looking at a future with Omicron VOC-mediated vaccination breakthroughs across the world, inclusive of India.

## Data Availability Statement

All the consensus fasta for the samples included in this study is available at the GISAID-EpiCoV (https://www.gisaid.org/) under the submissions having sequence ID identifiers EPI_ISL_3394861 to EPI_ISL_3394916. Further inquiries can be directed to the corresponding authors.

## Ethics Statement

The studies involving human participants were reviewed and approved by CSIR-IGIB’s Human Ethics Committee Clearance (Ref. No. CSIR-IGIB/IHEC/2020-21/02). The patients/participants provided their written informed consent to participate in this study.

## Author Contributions

AK, PM, and RM performed analysis. AK, PM, PD, SS, AS, and RP wrote the manuscript. PD, PC, SS, and AS performed the sequencing experiments. SB and BT provided the clinical samples and data. RP and SB designed, conceptualized, implemented, and coordinated the study, along with inferences of the results, and wrote the manuscript. PoD and VJ provided the clinical samples. VJ provided concept implementation. All authors contributed tothe article and approved the submitted version.

## Conflict of Interest

The authors declare that the research was conducted in the absence of any commercial or financial relationships that could be construed as a potential conflict of interest.

## Publisher’s Note

All claims expressed in this article are solely those of the authors and do not necessarily represent those of their affiliated organizations, or those of the publisher, the editors and the reviewers. Any product that may be evaluated in this article, or claim that may be made by its manufacturer, is not guaranteed or endorsed by the publisher.

## Supplementary Material

Supplementary Table 1 SARS-CoV-2 genome sequencing coverage, depth, sequencing platform, and lineage information sample-wise.

Supplementary Table 2 List of low- and high-frequency mutations in our cohort of VBTs with sub-phenotype distribution and literature references.

Supplementary Table 3 Change in physio-chemical properties of SARS-CoV-2 structural proteins due to the low- and high-frequency mutations in our cohort of VBTs.
