# Supplementary material for: Clinico-Genomic Analysis Reiterates Mild Symptoms Post-vaccination Breakthrough: Should We Focus on Low-Frequency Mutations?
Source: Front Microbiol. 2022 Mar 3;13:763169. doi: 10.3389/fmicb.2022.763169 (PMC8927057; doi:10.3389/fmicb.2022.763169)
Supplement: Supplementary Table 1 — SARS-CoV-2 genome sequencing coverage, depth, sequencing platform, and lineage information sample-wise. [file Data_Sheet_1.PDF]

Table S1: SARS-CoV-2 genome sequencing coverage, depth, sequencing platform and lineage information sample-wise.

Sample IDs	Sequencing platform	Sequencing depth	Coverage	Lineage
IGIB1130101V	Oxford nanopore Technology	1848.28	96.26	B.1.617.2 (Delta)
IGIB1130102915V	Oxford nanopore Technology	2696.91	99.54	B.1.617.2 (Delta)
IGIB1130103V	Oxford nanopore Technology	175.791	55.13	B.1.617.1 (Kappa)
IGIB11301042671V	Oxford nanopore Technology	2900.23	98.97	B.1.617.2 (Delta)
IGIB1130104V	Oxford nanopore Technology	647.471	89.38	B.1.1.7(Alpha, V1)
IGIB11301051274V	Oxford nanopore Technology	672.701	97.11	B.1.617.2 (Delta)
IGIB1130105V	Oxford nanopore Technology	1870.48	95.67	B.1.617.2 (Delta)
IGIB1130107V	Oxford nanopore Technology	179.615	66.24	B.1.1
IGIB1130108V	Oxford nanopore Technology	146.779	76.39	B.1.617.2 (Delta)
IGIB1130109V	Oxford nanopore Technology	248.276	86.95	B.1.617.2 (Delta)
IGIB1130110253V	Illumina MiSeq	63.93	99.6	B.1.617.2 (Delta)
IGIB1130110V	Oxford nanopore Technology	219.937	84	B.1.617.2 (Delta)
IGIB1130111V	Oxford nanopore Technology	1300.22	98.19	B.1.617.2 (Delta)
IGIB1130112885V	Illumina MiSeq	118.78	99.65	B.1.617.2 (Delta)
IGIB1130113V	Oxford nanopore Technology	2255.35	98.99	B.1.617.2 (Delta)
IGIB1130114V	Oxford nanopore Technology	247.087	87.55	B.1.617.2 (Delta)
IGIB1130125391V	Illumina MiSeq	11.48	95.22	B.1.1.7(Alpha, V1)

IGIB1130149231V	Illumina MiSeq	6532.53	99.87	B.1.617.2 (Delta)
IGIB1130151752V	Oxford nanopore Technology	918.302	97.2	B.1.617.2 (Delta)
IGIB113015915V	Illumina MiSeq	5337.44	99.83	B.1.617.2 (Delta)
IGIB1130164306V	Illumina MiSeq	7162.3	99.85	B.1.617.2 (Delta)
IGIB1130182572V	Oxford nanopore Technology	734.492	95.85	B.1.617.2 (Delta)
IGIB1130188012V	Oxford nanopore Technology	607.81	66.19	B.1.617.2 (Delta)
IGIB113023885V	Oxford nanopore Technology	1168.39	98.21	B.1.617.2 (Delta)
IGIB1130248022V	Oxford nanopore Technology	3049.66	98.63	B.1.617.2 (Delta)
IGIB1130251408V	Oxford nanopore Technology	150.641	78.81	B.1.617.2 (Delta)
IGIB1130263237V	Illumina MiSeq	2279.05	99.77	B.1.617.2 (Delta)
IGIB1130265694V	Illumina MiSeq	112.19	99.68	B.1.617.2 (Delta)
IGIB1130270387V	Illumina MiSeq	204.52	99.71	B.1.617.2 (Delta)
IGIB1130298132V	Oxford nanopore Technology	564.723	95.12	B.1.617.2 (Delta)
IGIB1130300073V	Illumina MiSeq	138.65	99.71	B.1.617.2 (Delta)
IGIB1130310652V	Illumina MiSeq	20.25	94.14	B.1.617.2 (Delta)
IGIB113031638V	Illumina MiSeq	27.76	99.12	B.1.617.2 (Delta)
IGIB1130331413V	Oxford nanopore Technology	83.6151	68.15	B.1.617.2 (Delta)
IGIB1130359925V	Oxford nanopore Technology	1245.23	97.09	B.1.617.2 (Delta)
IGIB1130368600V	Illumina MiSeq	249.82	99.71	B.1.617.2 (Delta)
IGIB1130370238V	Oxford nanopore	427.181	93.26	B.1.617.2

	Technology			(Delta)
IGIB1130371022V	Oxford nanopore Technology	2027.14	97.97	B.1.617.2 (Delta)
IGIB1130375373V	Illumina MiSeq	1066.43	99.72	B.1.617.2 (Delta)
IGIB1130390681V	Illumina MiSeq	2112.92	99.74	B.1.617.2 (Delta)
IGIB1130412732V	Oxford nanopore Technology	269.84	55.64	B.1.617.2 (Delta)
IGIB1130433112V	Illumina MiSeq	159.5	99.65	B.1.617.2 (Delta)
IGIB1130447004V	Illumina MiSeq	181.79	99.71	B.1.617.2 (Delta)
IGIB1130482058V	Oxford nanopore Technology	396.25	59.76	B.1.617.2 (Delta)
IGIB1130493051V	Oxford nanopore Technology	1600.3	84.66	B.1.617.2 (Delta)
IGIB113050041V	Oxford nanopore Technology	1618.99	82.56	B.1.617.2 (Delta)
IGIB1130519238V	Oxford nanopore Technology	745.44	66.84	B.1.617.2 (Delta)
IGIB1130527553V	Oxford nanopore Technology	872.63	72.13	B.1.617.2 (Delta)
IGIB1130534678V	Oxford nanopore Technology	151.2	50.75	B.1.617.2 (Delta)
IGIB113059193V	Oxford nanopore Technology	1817.31	97.65	B.1.617.2 (Delta)
IGIB1130751585V	Illumina MiSeq	6034.68	99.84	B.1.617.2 (Delta)
IGIB1130797358V	Oxford nanopore Technology	260.659	78.33	B.1.617.2 (Delta)
IGIB1130797709V	Oxford nanopore Technology	148.43	59.43	B.1.617.2 (Delta)
IGIB1130810142V	Illumina MiSeq	80.16	99.52	B.1.617.2 (Delta)
IGIB113093V	Oxford nanopore Technology	1334.58	98.5	B.1.617.2 (Delta)
IGIB113094002V	Oxford nanopore Technology	1550.33	74.69	B.1.617.2 (Delta)

Table S2: List of low and high frequency mutations in our cohort of VBTs with sub-phenotype distribution and literature references.

Nucleotide change	Amino acid change	Mild	Mild plus	P value	References
A23403G	D614G	27	29	-	(Thye et al., 2021; Socher et al., 2021; Mansbach et al., 2021; Zhang et al., 2021a; Yurkovetskiy et al., 2020)
C241T	C241T	25	27	0.94	(Mercatelli and Giorgi, 2020; Kozlovskaya et al., 2020; Luo et al., 2021; Xi et al., 2021)
G210T	G210T	24	27	0.58	(Xu et al., 2021; Kozlovskaya et al., 2020)
C14408T	P314L	23	24	0.8	(Githinji et al., 2021; Xu et al., 2021; Kozlovskaya et al., 2020)
C25469T	S26L	21	23	0.88	(Azad and Khan, 2021)
T26767C	I82T/S	21	22	0.86	(Schumann et al., 2021)
C16466T	P1000L	20	22	0.87	(Yadav et al., 2021)
G28881T	R203K	20	21	0.88	(Wu et al., 2021a; Zhao et al., 2021; Franco-Muñoz et al., 2020; Leary et al., 2021; Toyoshima et al., 2020)
A28461G	D63G	20	19	0.48	(Yang, 2021b; Sakai-Tagawa et al., 2021; Yang, 2021a; Shrivastava et al., 2021)
G24410A	D950N	20	18	0.33	(Sanches et al., 2021; Liu et al., 2021a; Davis et al., 2021; Zhang et al., 2021b; Lu et al.,

					2021)
C3037T	C3037T	17	20	0.63	(Justo Arevalo et al., 2021; Yuan et al., 2021; Cheng et al., 2021; Nguyen et al., 2020)
T22917G	L425R	17	20	0.63	(Alizon et al., 2021; Ahmad, 2021; Cosar et al., 2021)
C22995A	T478K	17	20	0.63	(Di Giacomo et al., 2021; Cherian et al., 2021)
C21618G	T19R	15	21	0.3	(Planas et al., 2021)
G29402T	D377Y	14	20	0.19	(Tu et al., 2021)
AGATTTC28247A	DF118-	16	17	0.96	
C23604G	P681R	13	17	0.43	(Saito et al., 2021; Cherian et al., 2021)
G29742T	G29742T	14	15	0.99	
C10029T	T3255I	13	14	0.99	(Kannan et al., 2021)
A11201G	T3646A	11	15	0.41	
ATACATG21764A	HV69/70-	1	0	-	(Kimura et al., 2021; Shamier et al., 2021; Gupta et al., 2021)
T24506G	S982A	1	0	-	(Wu et al., 2021b; Tada et al., 2021; Wang et al., 2021b; Xia et al., 2021; Yang et al., 2021)
G24914C	D1118H	1	0	-	(Xia et al., 2021; Liu et al., 2021b; Collier et al., 2021; Gupta et al., 2021; Wang et al., 2021b; Rajah et al., 2021)
C3267T	T1001I	1	1	0.95	
C5388A	A1708D	1	1	0.95	
A23063T	N501Y	1	1	0.95	(Tu et al., 2021; Colson et al., 2021; Liu et al., 2021c, 2021b)
C23271A	A570D	1	1	0.95	

A28111G	Y73C	1	1	0.95	(Singh et al., 2021; Akbulut, 2021)
G28280C	D3L	1	0	-	
C913T	C913T	1	1	0.95	
C344T	L27F	2	0	-	
C13620T	D51D	1	1	0.95	(Voss et al., 2021)
C14262T	D265D	0	2	-	
C14790T	I441I	1	1	0.95	
C15240T	N591N	1	1	0.95	(Ishikawa et al., 2021)
C25339T	D1259D	1	1	0.95	
C21855T	S98F	1	0	-	(Pulakuntla et al., 2021)
G21974C	D138Y	1	0	-	(Gong et al., 2021; Wang et al., 2021a; Salleh et al., 2021)
G25218T	G1219V	1	0	-	(Colson et al., 2021)
C25350T	P1263L	1	0	-	(Li et al., 2020; Mishra et al., 2021; Qi et al., 2021)
A28295G	N7D	1	0	-	
G28703T	D144H	0	1	-	
C29358T	T362I	0	1	-	(Rahman et al., 2021)
C6573T	S2103F	1	2	0.59	
G12940T	V4225V	1	2	0.59	
C17135T	P1223L	1	2	0.59	
C23525T	H655Y	3	0	-	(de Oliveira et al., 2021; Szemiel et al., 2021; Dejnirattisai et al., 2021; Colson et al., 2021; Vanderheiden et al., 2021)
C29738T	C29738T	1	1	0.95	

Table S3: Change in physio-chemical properties of SARS-CoV-2 structural proteins due to the low and high frequency mutations in our cohort of VBTs.

	Mutated peptide physicochemical properties					
**Physiological property**	**Wild type Nucleocapsid protein**	**Nucleocapsid protein D144H**	**Nucleocapsid protein N7D**	**Nucleocapsid protein A251V**	**Nucleocapsid protein R203K**	**Nucleocapsid protein T362I**

		**mutation**	**mutation**	**mutation**	**mutation**	**mutation**
**Number of residues:**	419	419	419	419	419	419
**Molecular weight:**	45625.17 g/mol	45647.22 g/mol	45626.15 g/mol	45653.22 g/mol	45597.15 g/mol	45637.22 g/mol
**Extinction coefficient:**	42530 M-1cm-1	42530 M-1cm-1	42530 M-1cm-1	42530 M-1cm-1	42530 M-1cm-1	42530 M-1cm-1
**Iso-electric point:**	pH 10.58	pH 10.62	pH 10.54	pH 10.58	pH 10.56	pH 10.58
**Net charge at pH 7:**	24.4	**25.5**	**23.4**	24.4	24.4	24.4
**Estimated solubility:**	Good water solubility	Good water solubility	Good water solubility	Good water solubility	Good water solubility	Good water solubility
**Physiological property**	**Wild type Spike protein**	**Spike protein D138Y mutation**	**Spike protein H655Y mutation**	**Spike protein S98F mutation**	**Spike protein G1219V mutation**	**Spike protein P1263L mutation**
**Number of residues:**	1273	1273	1273	1273	1273	1273
**Molecular weight:**	141176.94 g/mol	141225.02 g/mol	141202.97 g/mol	141237.04 g/mol	141219.02 g/mol	141192.98 g/mol
**Extinction coefficient:**	137400 M-1cm-1	138680 M-1cm-1	138680 M-1cm-1	137400 M-1cm-1	137400 M-1cm-1	137401 M-1cm-1
**Iso-electric point:**	pH 6.21	pH 6.3	pH 6.17	pH 6.21	pH 6.21	pH 6.21
**Net charge at pH 7:**	-8	**-7**	**-8.1**	-8	-8	-8
**Estimated solubility:**	Poor water solubility	Poor water solubility	Poor water solubility	Poor water solubility	Poor water solubility	Poor water solubility

References

Ahmad, L. (2021). Implication of SARS-CoV-2 Immune Escape Spike Variants on Secondary and Vaccine Breakthrough Infections. *Front. Immunol.* 12, 742167. doi:10.3389/fimmu.2021.742167.

Akbulut, E. (2021). Changes in Interaction Between Accessory Protein 8 and IL-17RA in UK Isolates Caused by Mutations in the SARS-CoV-2 Open Reading Frame 8. *International Journal of Computational and Experimental Science and Engineering*. doi:10.22399/ijcesen.935624.

Alizon, S., Haim-Boukobza, S., Foulongne, V., Verdurme, L., Trombert-Paolantoni, S., Lecorche, E., Roquebert, B., and Sofonea, M. T. (2021). Rapid spread of the SARS-CoV-2 δ variant in French regions in June 2021. *medRxiv*. doi:10.1101/2021.06.16.21259052.

Azad, G. K., and Khan, P. K. (2021). Variations in Orf3a protein of SARS-CoV-2 alter its structure and function. *Biochem. Biophys. Rep.* 26, 100933. doi:10.1016/j.bbrep.2021.100933.

Cheng, L., Han, X., Zhu, Z., Qi, C., Wang, P., and Zhang, X. (2021). Functional alterations caused by mutations reflect evolutionary trends of SARS-CoV-2. *Brief. Bioinformatics*. doi:10.1093/bib/bbab042.

Cherian, S., Potdar, V., Jadhav, S., Yadav, P., Gupta, N., Das, M., Rakshit, P., Singh, S., Abraham, P., Panda, S., et al. (2021). SARS-CoV-2 Spike Mutations, L452R, T478K, E484Q and P681R, in the Second Wave of COVID-19 in Maharashtra, India. *Microorganisms* 9. doi:10.3390/microorganisms9071542.

Collier, D. A., De Marco, A., Ferreira, I. A. T. M., Meng, B., Datir, R. P., Walls, A. C., Kemp, S. A., Bassi, J., Pinto, D., Silacci-Fregni, C., et al. (2021). Sensitivity of SARS-CoV-2 B.1.1.7 to mRNA vaccine-elicited antibodies. *Nature* 593, 136–141. doi:10.1038/s41586-021-03412-7.

Colson, P., Levasseur, A., Delerce, J., Pinault, L., Dudouet, P., Devaux, C., Fournier, P.-E., La Scola, B., Lagier, J.-C., and Raoult, D. (2021). Spreading of a new SARS-CoV-2 N501Y spike variant in a new lineage. *Clin. Microbiol. Infect.* 27, 1352.e1-1352.e5. doi:10.1016/j.cmi.2021.05.006.

Cosar, B., Karagulleoglu, Z. Y., Unal, S., Ince, A. T., Uncuoglu, D. B., Tuncer, G., Kilinc, B. R., Ozkan, Y. E., Ozkoc, H. C., Demir, I. N., et al. (2021). SARS-CoV-2 Mutations and their Viral Variants. *Cytokine Growth Factor Rev.* doi:10.1016/j.cytogfr.2021.06.001.

Davis, C., Logan, N., Tyson, G., Orton, R., Harvey, W. T., Perkins, J. S., Mollett, G., Blacow, R. M., COVID-19 Genomics UK (COG-UK) Consortium, Peacock, T. P., et al. (2021). Reduced neutralisation of the Delta (B.1.617.2) SARS-CoV-2 variant of concern following vaccination. *PLoS Pathog.* 17, e1010022. doi:10.1371/journal.ppat.1010022.

Dejnirattisai, W., Zhou, D., Supasa, P., Liu, C., Mentzer, A. J., Ginn, H. M., Zhao, Y., Duyvesteyn, H. M. E., Tuekprakhon, A., Nutalai, R., et al. (2021). Antibody evasion by the P.1 strain of SARS-CoV-2. *Cell* 184, 2939-2954.e9. doi:10.1016/j.cell.2021.03.055.

Di Giacomo, S., Mercatelli, D., Rakhimov, A., and Giorgi, F. M. (2021). Preliminary report on severe acute respiratory syndrome coronavirus 2 (SARS-CoV-2) Spike mutation T478K. *J. Med. Virol.* 93, 5638–5643. doi:10.1002/jmv.27062.

Franco-Muñoz, C., Álvarez-Díaz, D. A., Laiton-Donato, K., Wiesner, M., Escandón, P., Usme-Ciro, J. A., Franco-Sierra, N. D., Flórez-Sánchez, A. C., Gómez-Rangel, S., Rodríguez-Calderon, L. D., et al. (2020). Substitutions in Spike and Nucleocapsid proteins of SARS-CoV-2 circulating in South America. *Infect. Genet. Evol.* 85, 104557. doi:10.1016/j.meegid.2020.104557.

Githinji, G., de Laurent, Z. R., Mohammed, K. S., Omuoyo, D. O., Macharia, P. M., Morobe, J. M., Otieno, E., Kinyanjui, S. M., Agweyu, A., Maitha, E., et al. (2021). Tracking the introduction and spread of SARS-CoV-2 in coastal Kenya. *Nat. Commun.* 12, 4809. doi:10.1038/s41467-021-25137-x.

Gong, S. Y., Chatterjee, D., Richard, J., Prévost, J., Tauzin, A., Gasser, R., Bo, Y., Vézina, D., Goyette, G., Gendron-Lepage, G., et al. (2021). Contribution of single mutations to selected SARS-CoV-2 emerging variants spike antigenicity. *Virology* 563, 134–145. doi:10.1016/j.virol.2021.09.001.

Gupta, R., Kemp, S., Harvey, W., Lytras, S., Carabelli, A., and Robertson, D. (2021). Recurrent independent emergence and transmission of SARS-CoV-2 Spike amino acid H69/V70 deletions. *Res. Sq.* doi:10.21203/rs.3.rs-136937/v1.

Ishikawa, F., Udaka, Y., Oyamada, H., Ishino, K., Tokimatsu, I., Sagara, H., and Kiuchi, Y. (2021). Genetic epidemiology using whole genome sequencing and haplotype networks revealed the linkage of SARS-CoV-2 infection in nosocomial outbreak. *Infection Prevention in Practice*, 100190. doi:10.1016/j.infpip.2021.100190.

Justo Arevalo, S., Zapata Sifuentes, D., Huallpa, C. J., Landa Bianchi, G., Castillo Chávez, A., Garavito-Salini Casas, R., Uceda-Campos, G., and Pineda Chavarria, R. (2021). Global Geographic and Temporal Analysis of SARS-CoV-2 Haplotypes Normalized by COVID-19 Cases During the Pandemic. *Front. Microbiol.* 12, 612432. doi:10.3389/fmicb.2021.612432.

Kannan, S. R., Spratt, A. N., Cohen, A. R., Naqvi, S. H., Chand, H. S., Quinn, T. P., Lorson, C. L., Byrareddy, S. N., and Singh, K. (2021). Evolutionary analysis of the Delta and Delta Plus variants of the SARS-CoV-2 viruses. *J. Autoimmun.* 124, 102715. doi:10.1016/j.jaut.2021.102715.

Kimura, I., Kosugi, Y., Wu, J., Yamasoba, D., Butlertanaka, E. P., Tanaka, Y. L., Liu, Y., Shirakawa, K., Kazuma, Y., Nomura, R., et al. (2021). SARS-CoV-2 Lambda variant exhibits higher infectivity and immune resistance. *BioRxiv*. doi:10.1101/2021.07.28.454085.

Kozlovskaya, L., Piniaeva, A., Ignatyev, G., Selivanov, A., Shishova, A., Kovpak, A., Gordeychuk, I., Ivin, Y., Berestovskaya, A., Prokhortchouk, E., et al. (2020). Isolation and phylogenetic analysis of SARS-CoV-2 variants collected in Russia during the COVID-19 outbreak. *Int. J. Infect. Dis.* 99, 40–46. doi:10.1016/j.ijid.2020.07.024.

Leary, S., Gaudieri, S., Parker, M. D., Chopra, A., James, I., Pakala, S., Alves, E., John, M., Lindsey, B. B., Keeley, A. J., et al. (2021). Generation of a novel SARS-CoV-2 sub-genomic RNA due to the R203K/G204R variant in nucleocapsid: homologous recombination has potential to change SARS-CoV-2 at both protein and RNA level. *BioRxiv*. doi:10.1101/2020.04.10.029454.

Liu, C., Ginn, H. M., Dejnirattisai, W., Supasa, P., Wang, B., Tuekprakhon, A., Nutalai, R., Zhou, D., Mentzer, A. J., Zhao, Y., et al. (2021a). Reduced neutralization of SARS-CoV-2 B.1.617 by vaccine and convalescent serum. *Cell* 184, 4220-4236.e13. doi:10.1016/j.cell.2021.06.020.

Liu, Y., Liu, J., Plante, K. S., Plante, J. A., Xie, X., Zhang, X., Ku, Z., An, Z., Scharton, D., Schindewolf, C., et al. (2021b). The N501Y spike substitution enhances SARS-CoV-2 infection and transmission. *Nature*. doi:10.1038/s41586-021-04245-0.

Liu, Y., Liu, J., Plante, K. S., Plante, J. A., Xie, X., Zhang, X., Ku, Z., An, Z., Scharton, D., Schindewolf, C., et al. (2021c). The N501Y spike substitution enhances SARS-CoV-2 transmission. *BioRxiv*. doi:10.1101/2021.03.08.434499.

Li, Q., Wu, J., Nie, J., Zhang, L., Hao, H., Liu, S., Zhao, C., Zhang, Q., Liu, H., Nie, L., et al. (2020). The Impact of Mutations in SARS-CoV-2 Spike on Viral Infectivity and Antigenicity. *Cell* 182, 1284-1294.e9. doi:10.1016/j.cell.2020.07.012.

Luo, Y., Yu, F., Zhou, M., Liu, Y., Xia, B., Zhang, X., Liu, J., Zhang, J., Du, Y., Li, R., et al. (2021). Engineering a Reliable and Convenient SARS-CoV-2 Replicon System for Analysis of Viral RNA Synthesis and Screening of Antiviral Inhibitors. *MBio* 12. doi:10.1128/mBio.02754-20.

Lu, Y., Zhao, T., Lu, M., Zhang, Y., Yao, X., Wu, G., Dai, F., Zhang, F., and Zhang, G. (2021). The Analyses of High Infectivity Mechanism of SARS-CoV-2 and Its Variants. *COVID* 1, 666–673. doi:10.3390/covid1040054.

Mansbach, R. A., Chakraborty, S., Nguyen, K., Montefiori, D. C., Korber, B., and Gnanakaran, S. (2021). The SARS-CoV-2 Spike variant D614G favors an open conformational state. *Sci. Adv.* 7. doi:10.1126/sciadv.abf3671.

Mercatelli, D., and Giorgi, F. M. (2020). Geographic and Genomic Distribution of SARS-CoV-2 Mutations. *Front. Microbiol.* 11, 1800. doi:10.3389/fmicb.2020.01800.

Mishra, D., Suri, G. S., Kaur, G., and Tiwari, M. (2021). Comparative insight into the genomic landscape of SARS-CoV-2 and identification of mutations associated with the origin of infection and diversity. *J. Med. Virol.* 93, 2406–2419. doi:10.1002/jmv.26744.

Nguyen, T. T., Pham, T. N., Van, T. D., Nguyen, T. T., Nguyen, D. T. N., Le, H. N. M., Eden, J.-S., Rockett, R. J., Nguyen, T. T. H., Vu, B. T. N., et al. (2020). Genetic diversity of SARS-CoV-2 and clinical, epidemiological characteristics of COVID-19 patients in Hanoi, Vietnam. *PLoS ONE* 15, e0242537. doi:10.1371/journal.pone.0242537.

de Oliveira, T., Lutucuta, S., Nkengasong, J., Morais, J., Paula Paixao, J., Neto, Z., Afonso, P., Miranda, J., David, K., Ingles, L., et al. (2021). A novel variant of interest of SARS-CoV-2 with multiple spike mutations is identified from travel surveillance in Africa. *medRxiv*. doi:10.1101/2021.03.30.21254323.

Planas, D., Veyer, D., Baidaliuk, A., Staropoli, I., Guivel-Benhassine, F., Rajah, M. M., Planchais, C., Porrot, F., Robillard, N., Puech, J., et al. (2021). Reduced sensitivity of SARS-CoV-2 variant Delta to antibody neutralization. *Nature* 596, 276–280. doi:10.1038/s41586-021-03777-9.

Pulakuntla, S., Lokhande, K. B., Padmavathi, P., Pal, M., Swamy, K. V., Sadasivam, J., Singh, S. A., Aramgam, S. L., and Reddy, V. D. (2021). Mutational analysis in international isolates and drug repurposing against SARS-CoV-2 spike protein: molecular docking and simulation approach. *Virusdisease*, 1–13. doi:10.1007/s13337-021-00720-4.

Qi, H., Ma, M.-L., Jiang, H.-W., Ling, J.-Y., Chen, L.-Y., Zhang, H.-N., Lai, D.-Y., Li, Y., Guo, Z.-W., Hu, C.-S., et al. (2021). Systematic profiling of SARS-CoV-2-specific IgG epitopes at amino acid resolution. *Cell. Mol. Immunol.* 18, 1067–1069. doi:10.1038/s41423-021-00654-3.

Rahman, M. S., Islam, M. R., Alam, A. S. M. R. U., Islam, I., Hoque, M. N., Akter, S., Rahaman, M. M., Sultana, M., and Hossain, M. A. (2021). Evolutionary dynamics of SARS-CoV-2 nucleocapsid protein and its consequences. *J. Med. Virol.* 93, 2177–2195. doi:10.1002/jmv.26626.

Rajah, M. M., Hubert, M., Bishop, E., Saunders, N., Robinot, R., Grzelak, L., Planas, D., Dufloo, J., Gellenoncourt, S., Bongers, A., et al. (2021). SARS-CoV-2 Alpha, Beta, and Delta variants display enhanced Spike-mediated syncytia formation. *EMBO J.* 40, e108944. doi:10.15252/embj.2021108944.

Saito, A., Irie, T., Suzuki, R., Maemura, T., Nasser, H., Uriu, K., Kosugi, Y., Shirakawa, K., Sadamasu, K., Kimura, I., et al. (2021). Enhanced fusogenicity and pathogenicity of SARS-CoV-2 Delta P681R mutation. *Nature*. doi:10.1038/s41586-021-04266-9.

Sakai-Tagawa, Y., Yamayoshi, S., Halfmann, P. J., and Kawaoka, Y. (2021). Comparative Sensitivity of Rapid Antigen Tests for the Delta Variant (B.1.617.2) of SARS-CoV-2. *Viruses* 13. doi:10.3390/v13112183.

Salleh, M. Z., Derrick, J. P., and Deris, Z. Z. (2021). Structural Evaluation of the Spike Glycoprotein Variants on SARS-CoV-2 Transmission and Immune Evasion. *Int. J. Mol. Sci.* 22. doi:10.3390/ijms22147425.

Sanches, P. R. S., Charlie-Silva, I., Braz, H. L. B., Bittar, C., Freitas Calmon, M., Rahal, P., and Cilli, E. M. (2021). Recent advances in SARS-CoV-2 Spike protein and RBD mutations comparison between new variants Alpha (B.1.1.7, United Kingdom), Beta (B.1.351, South Africa), Gamma (P.1, Brazil) and Delta (B.1.617.2, India). *J. Virus Erad.* 7, 100054. doi:10.1016/j.jve.2021.100054.

Schumann, V.-F., Cuadrat, R., Wyler, E., Wurmus, R., Deter, A., Quedenau, C., Dohmen, J., Faxel, M., Borodina, T., Altmueller, J., et al. (2021). COVID-19 infection dynamics revealed by SARS-CoV-2 wastewater sequencing analysis and deconvolution. *medRxiv*. doi:10.1101/2021.11.30.21266952.

Shamier, M. C., Tostmann, A., Bogers, S., De Wilde, J., IJpelaar, J., Van Der Kleij, W., De Jager, H., Haagmans, B., Molenkamp, R., Oude Munnink, B., et al. (2021). Virological characteristics of SARS-CoV-2 vaccine breakthrough infections in health care workers. *medRxiv*. doi:10.1101/2021.08.20.21262158.

Shrivastava, S., Mhaske, S. T., Modak, M. S., Virkar, R. G., Pisal, S. S., Mishra, A. C., and Arankalle, V. A. (2021). Emergence of Two Distinct Variants of SARS-CoV-2 and Explosive Second Wave of COVID-19: An Experience From A Tertiary Care Hospital, Pune, India. *Res. Sq.* doi:10.21203/rs.3.rs-824960/v1.

Singh, J., Ehtesham, N. Z., Rahman, S. A., and Hasnain, S. E. (2021). Structure-function investigation of a new VUI-202012/01 SARS-CoV-2 variant. *BioRxiv*. doi:10.1101/2021.01.01.425028.

Socher, E., Conrad, M., Heger, L., Paulsen, F., Sticht, H., Zunke, F., and Arnold, P. (2021). Mutations in the B.1.1.7 SARS-CoV-2 Spike Protein Reduce Receptor-Binding Affinity and Induce a Flexible Link to the Fusion Peptide. *Biomedicines* 9. doi:10.3390/biomedicines9050525.

Szemiel, A. M., Merits, A., Orton, R. J., MacLean, O. A., Pinto, R. M., Wickenhagen, A., Lieber, G., Turnbull, M. L., Wang, S., Furnon, W., et al. (2021). In vitro selection of Remdesivir resistance suggests evolutionary predictability of SARS-CoV-2. *PLoS Pathog.* 17, e1009929. doi:10.1371/journal.ppat.1009929.

Tada, T., Dcosta, B. M., Samanovic, M. I., Herati, R. S., Cornelius, A., Zhou, H., Vaill, A., Kazmierski, W., Mulligan, M. J., and Landau, N. R. (2021). Convalescent-Phase Sera and Vaccine-Elicited Antibodies Largely Maintain Neutralizing Titer against Global SARS-CoV-2 Variant Spikes. *MBio* 12, e0069621. doi:10.1128/mBio.00696-21.

Thye, A. Y.-K., Law, J. W.-F., Pusparajah, P., Letchumanan, V., Chan, K.-G., and Lee, L.-H. (2021). Emerging SARS-CoV-2 Variants of Concern (VOCs): An Impending Global Crisis. *Biomedicines* 9. doi:10.3390/biomedicines9101303.

Toyoshima, Y., Nemoto, K., Matsumoto, S., Nakamura, Y., and Kiyotani, K. (2020). SARS-CoV-2 genomic variations associated with mortality rate of COVID-19. *J. Hum.*

Genet. 65, 1075–1082. doi:10.1038/s10038-020-0808-9.

Tu, H., Avenarius, M. R., Kubatko, L., Hunt, M., Pan, X., Ru, P., Garee, J., Thomas, K., Mohler, P., Pancholi, P., et al. (2021). Distinct Patterns of Emergence of SARS-CoV-2 Spike Variants including N501Y in Clinical Samples in Columbus Ohio. *BioRxiv*. doi:10.1101/2021.01.12.426407.

Vanderheiden, A., Thomas, J., Soung, A. L., Davis-Gardner, M. E., Floyd, K., Jin, F., Cowan, D. A., Pellegrini, K., Shi, P.-Y., Grakoui, A., et al. (2021). CCR2 Signaling Restricts SARS-CoV-2 Infection. *MBio*, e0274921. doi:10.1128/mBio.02749-21.

Voss, J. D., Skarzynski, M., McAuley, E. M., Maier, E. J., Gibbons, T., Fries, A. C., and Chapleau, R. R. (2021). Variants in SARS-CoV-2 associated with mild or severe outcome. *Evol. Med. Public Health* 9, 267–275. doi:10.1093/emph/eoab019.

Wang, P., Casner, R. G., Nair, M. S., Wang, M., Yu, J., Cerutti, G., Liu, L., Kwong, P. D., Huang, Y., Shapiro, L., et al. (2021a). Increased resistance of SARS-CoV-2 variant P.1 to antibody neutralization. *Cell Host Microbe* 29, 747-751.e4. doi:10.1016/j.chom.2021.04.007.

Wang, Y., Wu, J., Zhang, L., Zhang, Y., Wang, H., Ding, R., Nie, J., Li, Q., Liu, S., Yu, Y., et al. (2021b). The Infectivity and Antigenicity of Epidemic SARS-CoV-2 Variants in the United Kingdom. *Res. Sq.* doi:10.21203/rs.3.rs-153108/v1.

Wu, H., Xing, N., Meng, K., Fu, B., Xue, W., Dong, P., Tang, W., Xiao, Y., Liu, G., Luo, H., et al. (2021a). Nucleocapsid mutations R203K/G204R increase the infectivity, fitness, and virulence of SARS-CoV-2. *Cell Host Microbe*. doi:10.1016/j.chom.2021.11.005.

Wu, J., Zhang, L., Zhang, Y., Wang, H., Ding, R., Nie, J., Li, Q., Liu, S., Yu, Y., Yang, X., et al. (2021b). The Antigenicity of Epidemic SARS-CoV-2 Variants in the United Kingdom. *Front. Immunol.* 12, 687869. doi:10.3389/fimmu.2021.687869.

Xia, S., Wen, Z., Wang, L., Lan, Q., Jiao, F., Tai, L., Wang, Q., Sun, F., Jiang, S., Lu, L., et al. (2021). Structure-based evidence for the enhanced transmissibility of the dominant SARS-CoV-2 B.1.1.7 variant (Alpha). *Cell Discov.* 7, 109. doi:10.1038/s41421-021-00349-z.

Xi, B., Jiang, D., Li, S., Lon, J. R., Bai, Y., Lin, S., Hu, M., Meng, Y., Qu, Y., Huang, Y., et al. (2021). AutoVEM: An automated tool to real-time monitor epidemic trends and key mutations in SARS-CoV-2 evolution. *Comput. Struct. Biotechnol. J.* 19, 1976–1985. doi:10.1016/j.csbj.2021.04.002.

Xu, G., Li, Y., Zhang, S., Peng, H., Wang, Y., Li, D., Jin, T., He, Z., Tong, Y., Qi, C., et al. (2021). SARS-CoV-2 promotes RIPK1 activation to facilitate viral propagation. *Cell Res.* 31, 1230–1243. doi:10.1038/s41422-021-00578-7.

Yadav, P. D., Sahay, R. R., Sapkal, G., Nyayanit, D., Shete, A. M., Deshpande, G., Patil, D. Y., Gupta, N., Kumar, S., Abraham, P., et al. (2021). Comparable neutralization of

SARS-CoV-2 Delta AY.1 and Delta with individuals sera vaccinated with BBV152. *J. Travel Med.* doi:10.1093/jtm/taab154.

Yang, J., Zhang, P., Cheng, W. X., Lu, Y., Gang, W., and Ren, G. (2021). Exposing structural variations in SARS-CoV-2 evolution. *Sci. Rep.* 11, 22042. doi:10.1038/s41598-021-01650-3.

Yang, X.-J. (2021a). SARS-COV-2 δ variant drives the pandemic in India and Europe via two subvariants. *medRxiv*. doi:10.1101/2021.10.16.21265096.

Yang, X.-J. (2021b). SARS-COV-2 δ variant drives the pandemic in the USA through two subvariants. *Res. Sq.* doi:10.21203/rs.3.rs-986605/v1.

Yuan, F., Wang, L., Fang, Y., and Wang, L. (2021). Global SNP analysis of 11,183 SARS-CoV-2 strains reveals high genetic diversity. *Transbound. Emerg. Dis.* 68, 3288–3304. doi:10.1111/tbed.13931.

Yurkovetskiy, L., Wang, X., Pascal, K. E., Tomkins-Tinch, C., Nyalile, T. P., Wang, Y., Baum, A., Diehl, W. E., Dauphin, A., Carbone, C., et al. (2020). Structural and Functional Analysis of the D614G SARS-CoV-2 Spike Protein Variant. *Cell* 183, 739-751.e8. doi:10.1016/j.cell.2020.09.032.

Zhang, J., Cai, Y., Xiao, T., Lu, J., Peng, H., Sterling, S. M., Walsh, R. M., Rits-Volloch, S., Zhu, H., Woosley, A. N., et al. (2021a). Structural impact on SARS-CoV-2 spike protein by D614G substitution. *Science* 372, 525–530. doi:10.1126/science.abf2303.

Zhang, J., Xiao, T., Cai, Y., Lavine, C. L., Peng, H., Zhu, H., Anand, K., Tong, P., Gautam, A., Mayer, M. L., et al. (2021b). Membrane fusion and immune evasion by the spike protein of SARS-CoV-2 Delta variant. *Science* 374, 1353–1360. doi:10.1126/science.abl9463.

Zhao, M., Yu, Y., Sun, L.-M., Xing, J.-Q., Li, T., Zhu, Y., Wang, M., Yu, Y., Xue, W., Xia, T., et al. (2021). GCG inhibits SARS-CoV-2 replication by disrupting the liquid phase condensation of its nucleocapsid protein. *Nat. Commun.* 12, 2114. doi:10.1038/s41467-021-22297-8.
